# Photo-Induced Doping in a Graphene Field-Effect Transistor with Inkjet-Printed Organic Semiconducting Molecules

**DOI:** 10.3390/nano9121753

**Published:** 2019-12-10

**Authors:** Nikita Nekrasov, Dmitry Kireev, Nejra Omerović, Aleksei Emelianov, Ivan Bobrinetskiy

**Affiliations:** 1 National Research University of Electronic Technology, 124498 Moscow, Russia; 8141147@gmail.com (N.N.); emmsowton@gmail.com (A.E.); 2 Department of Electrical and Computer Engineering, The University of Texas at Austin, Austin, TX 78758, USA; kirdmitry@gmail.com; 3 BioSense Institute-Research and Development Institute for Information Technologies in Biosystems, University of Novi Sad, 21000 Novi Sad, Serbia; nejra@biosense.rs; 4 P.N. Lebedev Physical Institute of the Russian Academy of Sciences, 119991 Moscow, Russia

**Keywords:** CVD graphene, field-effect transistor, in-plane junction, non-covalent functionalization, semiconducting organic molecules, inkjet printing, photoresponse

## Abstract

In this work, we report a novel method of maskless doping of a graphene channel in a field-effect transistor configuration by local inkjet printing of organic semiconducting molecules. The graphene-based transistor was fabricated via large-scale technology, allowing for upscaling electronic device fabrication and lowering the device’s cost. The altering of the functionalization of graphene was performed through local inkjet printing of *N*,*N*′-Dihexyl-3,4,9,10-perylenedicarboximide (PDI-C6) semiconducting molecules’ ink. We demonstrated the high resolution (about 50 µm) and accurate printing of organic ink on bare chemical vapor deposited (CVD) graphene. PDI-C6 forms nanocrystals onto the graphene’s surface and transfers charges via π–π stacking to graphene. While the doping from organic molecules was compensated by oxygen molecules under normal conditions, we demonstrated the photoinduced current generation at the PDI-C6/graphene junction with ambient light, a 470 nm diode, and 532 nm laser sources. The local (in the scale of 1 µm) photoresponse of 0.5 A/W was demonstrated at a low laser power density. The methods we developed open the way for local functionalization of an on-chip array of graphene by inkjet printing of different semiconducting organic molecules for photonics and electronics.

## 1. Introduction

Hybrid organic/inorganic heterostructures have become highly engaging materials, providing novel properties in electrical conduction, optical responsivity, and flexibility, and generating novel technology for their production, paving the way for a new class of hybrid functional materials whose final properties can be selected by careful molecular design [1,2]. As far as graphene and other 2D structures are highly sensitive to the environment, the organic molecules, once they been adsorbed on a surface, they can significantly alter the electrical and optical properties by a doping effect [3]. Due to efficient charge transfer at the interface, the organic molecules can dramatically tune the Dirac point of graphene [4,5,6] and change the transport behaviour under light illumination, providing ultrasensitive and broadband light-detection capabilities [7,8]. In particular, perylene-based dyes have been implemented in many fields of 2D materials technology because of their outstanding physical and chemical properties [9]. Perylene diimide (PDI) chromophore derivatives have been successfully introduced for non-covalent functionalization of graphene in tasks of exfoliation, stabilization of graphene solutions, uniform decoration by other nanoparticles, thin film preparation, increasing the thermal properties of composites, drug delivery, and novel optoelectronic devices [10]. PDI interacts with graphene via π–π stacking with strong chemical doping by charge transfer that can be either *p*-type [11] or *n*-type [12] depending on radicals attached to PDI. The effective core-shell *p*-*n* heterojunction nanomaterial was demonstrated based on PDI derivatives and reduced graphene oxide as a field-effect phototransistor with high external quantum efficiency [13]. Despite high efficiency of the graphene-based semiconductor heterojunction for photonic applications, there are still challenging tasks in long-term reliability and durability, environmental-friendliness, and cost-effectiveness for machining technologies in large-scale production, which should be solved [14].

Organic molecules can be deposited either by thermal evaporation from solid-state or by a solution process. The former gives less impurity from the deposited organic crystals but demands vacuum equipment and the necessity of photolithography processes [2,6,8]. Solution-based methods allow maskless deposition of the molecules, for example, using inkjet printing, even for complex array structures [15,16]. Inkjet printing performs accurate positioning of down to picolitre drops of semiconducting organic materials with resolution in about the tens of micrometres [17]. Recently, this technique was used to develop printed products by manufacturing large-area organic electronics with comparable performances to the traditional methods [18]. Nevertheless, ink preparation is still a challenging task and must be tuned to each type of molecule and type of printer in use [19,20].

This work reports on the maskless method of hybrid photosensitive junction fabrication in a graphene field-effect transistor (GFET) based on the inkjet printing of semiconducting organic ink. While graphene serves as a conductive channel, the non-covalent functionalization with semiconducting organic molecules alters the optical sensitivity in normal conditions to the visible wavelengths. We showed the one-step technique of graphene-channel functionalization by PDI molecules and in-plane junction formation by local printing of organic inks. The suggested method of junction formation in GFET is a prospective approach for a non-covalent functionalization of an array of graphene-based transistors by different organic molecules for photonic and electronic device fabrication.

## 2. Materials and Methods

### 2.1. Graphene Growth and GFET Fabrication

GFETs were fabricated using a chemical vapor deposited (CVD), single-layer graphene. To assist in transfer, a thin layer of poly(methyl methacrylate) (PMMA) was spin-coated on top of the graphene/copper. A previously published, high-throughput transfer technique of graphene transfer was utilized in this work [21]. In a nutshell, the PMMA/graphene/copper stack was immersed into copper etchant (0.1 M ammonium persulfate) for 8–12 h, washed in a cascade of clean deionized water (DI) water, and then transferred onto the target wafer. To re-flow the PMMA and improve the graphene-to-substrate adhesion, we annealed the wafer at 150 °C for 10 min [22]. Afterward, the PMMA was dissolved in acetone, washed with isopropanol (IPA) and DI water, dried under nitrogen flow, and annealed at 350 °C in a N_2_ atmosphere. The graphene was then patterned via oxygen plasma etching (300 W, 200 sccm, 10 min). Using e-beam assisted evaporation, we deposited the 10 nm Ti and 100 nm Au metallization. The details of the fabrication process can also be found elsewhere [23]. Prior to the sensor assembly, the surface of GFETs was UV treated in the air for 4 min to remove the organic residuals and activate the carbon bonds.

### 2.2. Organic Semiconductors Ink Preparation

We used the following *N*,*N*′-Dihexyl-3,4,9,10-perylenedicarboximide (PDI-C6) (Sigma Aldrich, St. Louis, MO, USA) semiconducting organic molecules for ink preparation. The inks of 0.7 mg of organic molecules were prepared in 6 mL of toluene/IPA (50/50%) solution. Toluene (0.36 D) was used as a less polar solvent for PDI. As far as the viscosity of toluene is small (0.56 cP), we added IPA (2.04 cP) to prepare the ink. For ultrasound treatment of the ink, the ultrasound bath Bandelin Sonorex was used for 30 min. Nevertheless, PDI had sedimented, and the top layer of the solution was taken for inkjet cartridge filling. The ink was filtered using a filter with pore size of 200 nm (Chromafil CA-20/25, cellulose acetate) before loading to the cartridge.

### 2.3. Ink-Jet Printing

For inkjet printing, Dimatix Materials Printer DMP-3000 (Fujifilm, Tokyo, Japan) inkjet printer was used. The printer operates via piezoelectric jetting cartridges with 21.5 µm nozzle diameters. In order to obtain a controllable process, basic printing parameters, such as waveform, frequency, jetting voltage, and drop spacing, were adjusted. For low-viscous ink (<2 cP), the waveform of pulse supplied on piezoelectric nozzles is the main parameter to control the formation of the stable droplets. To ensure stable conditions during the process and proper layer formation, the substrate and the cartridge worked at room temperature. All the experiments with organic inkjet printing were performed in cleanroom facilities.

### 2.4. Graphene FET Characterization

We measured the electrical characteristics via a semiconductor parameter analyser, IPPP 1/5 (MNIPI, Minsk, Belarus). For transfer current-voltage characteristic measurements, we used the liquid gate configuration with phosphate-buffered saline and AgCl reference electrode. Optical characterization was performed with HRM-300 (Huvitz BD, Dongan, Republic of Korea) with 5×–50× objectives. Raman spectra were recorded on a Centaur HR Raman spectrometer (Nanoscan Technology, Dolgoprudnyy, Russia) with a 100× objective at 532 nm wavelength (Cobolt, Solna, Sweden) with a beam spot of ≈1 μm^2^ and laser power of 0.5 mW. A Solver Pro atomic force microscope (AFM) (NT-MDT, Moscow, Russia) was used to study the morphology of pristine graphene, and later, its modification with organic molecules.

### 2.5. Photoresponse Measurements

To evaluate the photocurrent response across the GFET, we used the same Raman laser with 0.5 mW maximum power delivered through 50× fixed lens while the sample moved on the motorized stage. The steps between measured points were 1 µm with 20 s delay between measurements.

To conduct the photocurrent measurements, 470 nm light-emitting diode (LED) was used (Thorlabs, Newton, NJ, USA). An LED was placed at a distance of 10 cm above the structures. The experiments were performed in the room light and in the light-protected box with and without LED illumination.

## 3. Results and Discussion

### 3.1. Inkjet Printing of Organic Molecules

#### 3.1.1. Non-Covalent Functionalization of Graphene by an Array of Organic Molecules

**Figure 1:**
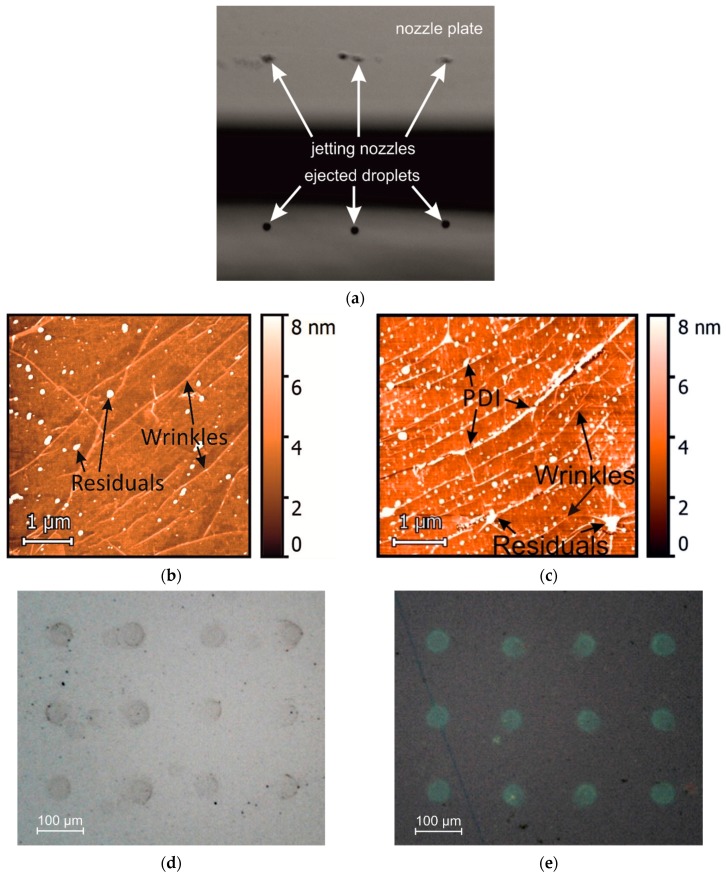
Inkjet printing of semiconducting organic’s ink. (**a**) Optical image of PDI-C6 ink drops jetting from the printer’s nozzles. (**b**,**c**) atomic force microscope (AFM) images zoom into the bare graphene surface (**b**) and inkjet-printed PDI-drop-film on the graphene surface (**c**). The scale bar is 1 µm. (**d**,**e**) The optical images of an array of PDI dots inkjet-printed on a silicon substrate (**d**) and graphene on Si/SiO_2_ (**e**). The scale bar is 100 µm.

The PDI-based ink was prepared in order to fulfil the demands for viscosity and surface tension for stable printing (Figure 1a) and to achieve regular film deposition of semiconducting molecules onto the graphene surface. The atomic force microscopy (Figure 1b,c) revealed that in addition to molecular layer deposition on the bare graphene surface, the PDI-C6 formed the nanocrystals with height of about 5 ± 1 nm. Molecules form an island-like film structure on graphene, attaching dominantly to the irregularities on the surface. IPA was used as an excellent ink matrix, providing both high viscosity and low surface tension (23 mN/m). Toluene was found as the best solvent for a stable solution of PDI-C6. Moreover, the toluene/IPA mixture should decrease the negative “coffee-ring” effect, which creates a ring-like morphology after the droplet dries on the substrate due to the accumulation of the solutes during the solvent evaporation [15,20]. We printed the solution on silicon substrate and CVD graphene and found a “coffee-ring” and irregular shape of a drop on pure Si substrate (Figure 1d) because of high surface tension. Graphene was pretreated with a UV lamp for 4 min, which significantly decreased the surface tension, and the drops had perfect circular-shapes on their surfaces (Figure 1e).

**Figure 2:**
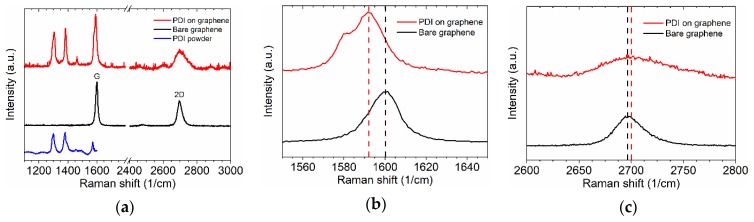
Raman spectra of bare graphene on Si/SiO_2_, PDI powder, and PDI film on graphene. (**a**) Overview of the Raman spectra in the relevant frequency range, between 1100 and 3000 cm^−1^; (**b**,**c**) a close-up view of the frequency range revealing the changes of the G peak (**b**) and the 2D peak (**c**) after the molecules’ deposition.

Before the characterization of printed layers, the post-processing was done by temperature annealing on a hot plate at 150 °C for 15 min to evaporate the solvent matrix. The results of the Raman measurements for PDI-C6 modified graphene are presented in Figure 2a. PDI-specific peaks could be determined precisely in the spectra of both PDI-C6 samples (PDI-C6 powder and PDI-C6 layer on graphene), their locations being determined as 1305 and 1383 cm^−1^ (in-plane ring “breathing”), 1354 cm^−1^ (out-of-plane C–C stretching), 1462 cm^−1^ (ring deformation), and 1581 cm^−1^ (in-plane C–C stretching), respectively [24,25]. As far as PDI-C6 molecule consists of seven benzene rings, it has the vibration mode both for in-plane and out of plane C–C, which are clearly observed via Raman for both powder and thin film on the graphene surface that can interfere the D and G-bands of graphene. For the bare graphene, the G peak was found at 1600 cm^−1^ and the 2D peak at 2700 cm^−1^, respectively, whereas for the “PDI on graphene” sample, their locations were at 1592 and 2699 cm^−1^. The charges in deposited organic molecules induced doping of graphene, and we observed a clear redshift of the G band up to 8 cm^−1^ [26] (Figure 2b). The doping was also confirmed by the broadening of the 2D band of graphene (Figure 2c) [6]. PDI-C6 is an electron-accepting material typically used in organic light emitted diodes and solar cells [12,27]. The Raman intensity ratio I_2D_/I_G_ of the bare graphene (0.5) and the one for graphene covered with a PDI adsorbate layer (0.3) further confirm the π–π stacking of PDI on graphene, and the chemical doping of graphene as well [28]. The slight shift of peaks for PDI-C6 after deposition on graphene can be explained by interaction of organic molecules with a graphene surface. It should be noted that in the presence of graphene, the luminescence background observed for PDI powder is quenched, which allows one to measure a clear Raman signal on emitting molecules [29]. The emission background is eliminated by charge transfers from PDI to graphene [25].

#### 3.1.2. Formation of a Heterojunction in GFET via Local Organic Molecular Printing

**Figure 3:**
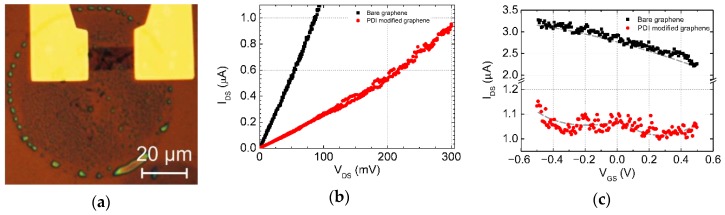
Optical image of a single drop of PDI ink printed on the graphene channel (**a**). The change in current–voltage characteristics before and after PDI ink printing: I versus V_SD_ (**b**); I versus V_G_ (**c**).

We applied the novel method for the inkjet printing of PDI-C6 on the graphene channel in field-effect transistor configuration. Figure 3a shows the optical image of PDI drop printed on the GFET channel before thermal annealing. One can still notice the presence of “coffee ring” effect on Si/SiO_2_ substrate after single droplet printing. The film deposited inside the drop is not uniform because of differences in evaporation temperatures of several components of the ink that led to clustering during droplet drying. We observed an increase in resistance of the graphene channel of about 2–3 times (Figure 3b). The bare graphene was highly *p*-type doped, as was demonstrated for both liquid gate and bottom gate measurements. Thus, for liquid gate measurement, the Dirac point for bare graphene was not reached because of the limitation of gate current leakage during the experiments (Figure 3c). After the deposition of PDI, we observed the change in slope and form of the transfer characteristic. The double Dirac voltages observed at −0.2 and 0.3 V correspond to *n*-type and *p*-type doping, respectively [2,8], whereas *n*-type doping of graphene originates from high electron transfer from the PDI layer. However, the effect was still weak due to the decrease of the main charge carriers and rise of the noise level because of not-covalently absorbed organic molecules.

Designed inkjet printing produces a molecular-level-thickness film on graphene, but the doping effect can be altered by oxygen molecules in the air, which are responsible for strong initial *p*-type doping of graphene in an ambient environment [5,30]. This, however, contradicts the doping effect observed on Raman spectra for coated region of the graphene channel. To prove the doping effect from molecules, we applied the local optical probing [8] on the fabricated GFET.

### 3.2. Photoresponse in Doped Graphene/Organic Nanostructures

**Figure 4:**
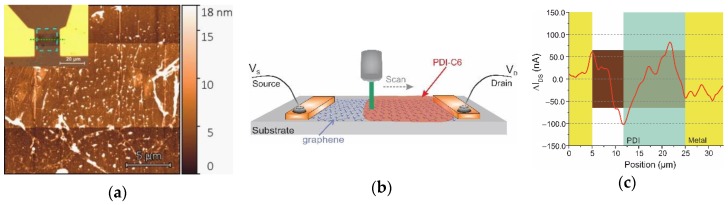
(**a**) AFM image of the graphene channel of FET with the right part modified by PDI. Insert: the optical image of the device with a dotted square marking the area of AFM image. (**b**) Schematic illustration of the GFET device with inkjet-printed PDI layer and the optical setup. (**c**) Line profile of the photocurrent response measured at 60 µW 532 nm wavelength along the dashed green line in ((a), insert). The yellow background indicates electrode positions, violet—graphene, and the green is the PDI-C6 film.

We studied the photoresponse in in-plane junction made by the local deposition of PDI-C6 ink only on the part of the graphene channel (Figure 4a). The film consisted of a thin PDI-C6 molecular island-like layer and some aggregates in agreement with the deposition of the ink on the bare graphene, as shown in Figure 2b. There is still a trace of the “coffee ring” effect that can give a distinct and rather narrow edge of the drop deposited on graphene. Photoresponse profiling was performed using Raman laser system with connected semiconductor parameter analyser to the transistors while the sample was moved across the focused laser beam (Figure 4b).

When a laser beam irradiates the graphene-contact areas, we observe the obvious increase of photocurrent at the barrier formed between graphene and metal electrode because of local doping at the interface of electrode and formation of space charge region [31] (Figure 4c). Photocurrent has opposite signs at different contacts, and there is a weak photocurrent generation in biased, bare graphene air [32]. Similar modulation of Fermi level in graphene-based Schottky barrier was also observed for graphene/Si photosensitive junctions, which is mainly caused by band structure of graphene and its low electron densities of states close to the Dirac point [33].

We noticed the strong increase of photocurrent at the junction of bare graphene and PDI-C6/graphene, indicating the presence of inhomogeneous profile of carrier density in graphene induced by an alignment of the Fermi level in differently doped regions [34]. The photocurrent that was generated had a triangular shape as a function of a distance from the junction with a distance about 4.5 µm and 6 µm for bare graphene and PDI-C6 parts, respectively. This area was much broader than the edge of the junction observed in Figure 4a, and exceeded, by more than twice, the photocurrent profile measure for hot charge carriers generated in bare exfoliated graphene [35], but was in the same range as CVD graphene modified by organic molecules [2,34]. Impurities and defects can act as trap centres in graphene, which greatly prolongs the carrier lifetime [36,37]. Thus, the presence of such traps as organic molecules can increase spatial distribution far from the location of the junction. This is clearly seen in Figure 4c by the broad photocurrent profile for both the PDI-C6/graphene junction and PDI-C6/graphene/gold interface because absorbed molecules increase the number of charge traps near graphene. The photocurrent generated in the junction is two times higher than in graphene/metal interfaces, that is, only 30% less than for the *p*–*n* junction created by organic interface doping [34], indicating the suggested inkjet printing efficiency for potential photodetector applications (Table 1).

The photoresponse in graphene/organic interface can have either photovoltaic [8] or photothermoelectric nature [34]. PDI-C6 molecules deposited onto graphene donate the channel with electrons, converting it to an *n*-type region. On the other hand, the significant charge transfer from the molecules occurred only under light irradiation, while there was no real *n*-type doping observed for modified graphene transistor in the dark (Figure 3c), which indicates that the photovoltaic effect takes precedence over the thermoelectric effect in this configuration with a low bias regime [44].

**Figure 5:**
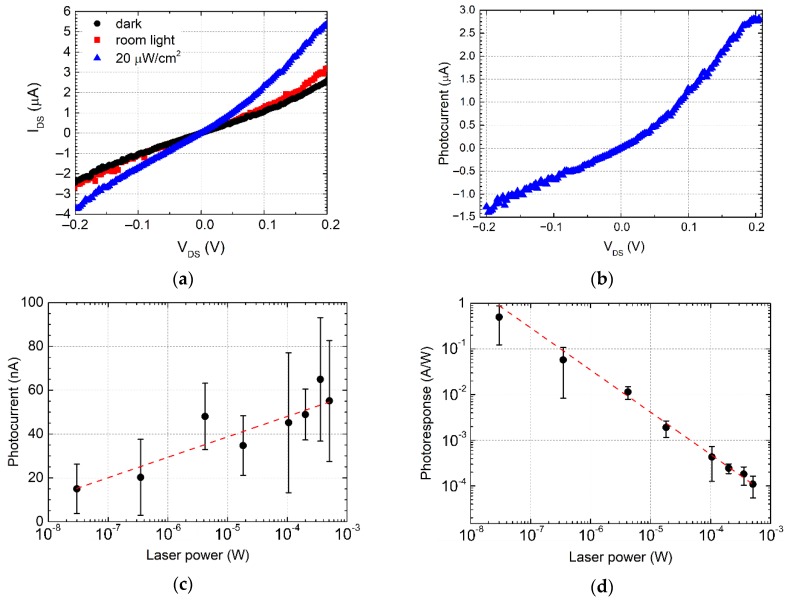
Photoresponse in PDI-C6/graphene junction. (**a**) Drain current versus bias voltage with and without light illumination: in the dark (black), room light (red), and 470 nm diode light (blue). (**b**) Photocurrent as a function of source-drain voltage measured for 470 nm diode light illumination. (**c**,**d**) Variation of photocurrent (**c**) and responsivity (**d**) of the in-plane junction as a function of the incident laser power at 532 nm. The dashed red line is a guide to the eye.

To investigate the efficiency of the photodetectors, we performed characterization under an ambient and an LED light. The results are shown in Figure 5. The devices were illuminated with 35 µW/cm^2^ white room light and with 470 nm 20 µW/cm^2^ LED. In Figure 5a, we show that the in-plane junction structure slightly responds to the white light and has a noticeable photocurrent (*I*_ph_) when it is irradiated with a 470 nm light source. The output curves are non-linear after the organic molecules’ deposition due to the difference of barrier height at the interface of metal with bare graphene, and graphene modified by organic molecules [31]. The maximum responsivity (*R*) for 470 nm LED was calculated as *R* = *I*_ph_/*P* (where *P*—is an incident light power), which is equal to 0.15 A/W at 0.2 V bias voltage (Figure 5b).

Figure 5c,d shows the photocurrent and response of the samples to 532 nm Raman laser. We observed that for higher incident laser power, the photoresponse decreases, which can be explained by the saturation effect for charge carrier transport due to the small covered area by organic molecules. Also, the heating of molecules and graphene is possible for higher intensities leading to increasing the thermoelectric effect that can compensate the photovoltaic effect [34]. It should be noted that we implemented the same process of PDI inkjet printing on similar graphene structures but without UV treatment. We found very few organic molecules deposited and no photodoping effect for the majority of structures. We suggest that contaminants and organic residuals on the graphene surface increase the surface tension after transistor fabrication and its storage, which decreases the efficiency of inkjet printing. Short UV treatment provides gentle cleaning of the graphene surface from organic contaminations [45].

## 4. Conclusions

In conclusion, we have developed robust and scalable technology for the functionalization of arrays of graphene field-effect transistors by inject printing. We prepared the semiconducting organic ink and studied the deposition of organic molecules onto the graphene surface. We demonstrated the resolution of printing is consistent with current imaging techniques. This technology is very flexible and can provide the modification of an array of GFET on the same substrate with different organic molecules that can be tuned for specific wavelengths. The drastic changes in the electrical properties of functionalized graphene with organic molecules were shown. The in-plane photosensitive junction was developed based on partial coverage of the graphene channel. The junction is sensitive to visible light, with a photoresponse up to 0.5 A/W for 532 nm light at 30 nW light intensity. We suggested that the photovoltaic effect is responsible for photocurrent generation in a junction created by light-induced doping of graphene from organic molecules.

## Figures and Tables

**Table 1:** Comparison of the fabrication methods for photosensitive, hybrid, in-plane junctions in graphene.

Junction’s Material	Method	Min. Resolution, µm	Max. R (A·W^−1^)	Rev
FeCl_3_	cw laser	5	0.1·10^−3^	[38]
Oxygen groups	fs laser	1	0.1	[39]
Organic dye (rhodamine 6G)	drop cast	-	460	[40]
Graphene quantum dots	drop cast	-	1	[41]
Lithium enriched SU-8	Photolithography	2	0.025	[2]
Silane SAMs	e-beam lithography	10	0.03	[34]
P3HT:PCBM	Spin coating	-	~0.05	[42]
Perovskite	Spin coating	-	0.343	[43]
Rhodamine 6G	Photolithography	~1	-	[8]
PDI-C6	Inkjet printing	50	0.5	*This work*
